# Phenolic Compounds and Antioxidant, Antibacterial, and α-Amylase-/α-Glucosidase-Inhibitory Activities of Plant Species from the Genera *Persicaria*, *Polygonum*, and *Fallopia*

**DOI:** 10.3390/plants15213333

**Published:** 2026-10-28

**Authors:** Liubov Skrypnik, Anna Pavlova, Nikolay Belov, Arina Iureva

**Affiliations:** Institute of Natural Sciences, Immanuel Kant Baltic Federal University, 236040 Kaliningrad, Russia

**Keywords:** medicinal plants, chemotaxonomy, secondary metabolites, diabetes, raw material, Polygonaceae

## Abstract

The comparative analysis of extracts from eight closely related species belonging to the Polygonaceae family (*Persicaria lapathifolia*, *Persicaria hydropiper*, *Persicaria maculosa*, *Persicaria amphibia*, *Persicaria minor*, *Persicaria bistorta*, *Polygonum aviculare*, and *Fallopia convolvulus*) and growing under identical environmental conditions is presented in this study. The total contents of phenolic compounds, flavonoids, hydroxycinnamic acids, catechins, proanthocyanidins, and tannins were determined using spectrophotometric methods. The maximum concentrations of phenolic compounds were found in *P. maculosa* (up to 70 mg g^−1^); flavonoids in *P. hydropiper* (about 45 mg g^−1^); hydroxycinnamic acids in *P. minor*, *P. bistorta*, and *F. convolvulus* (11.7–15.5 mg g^−1^); catechins in *P. bistorta* and *P. aviculare* (6.2–6.4 mg g^−1^); proanthocyanidins in *P. bistorta* (about 3.6 mg g^−1^); and tannins in *P. hydropiper* and *P. maculosa* (5.8–6.5 mg g^−1^). The qualitative and quantitative composition of phenolic compounds was determined by high-performance liquid chromatography with diode-array detection (HPLC-DAD) by comparing retention times and UV spectra with those of the relevant standards. The predominance of flavonol derivatives (quercetin and kaempferol) over flavone derivatives in the species studied was revealed. *P. hydropiper*, *P. maculosa*, and *P. lapathifolia* were found to contain high concentrations of ellagic acid and its derivatives (about 15 mg g^−1^), which correlated with the higher antioxidant activity characteristic of the extracts from these species. Based on their inhibitory activity (exceeding 70%) against α-amylase and yeast α-glucosidase, *P. hydropiper*, *P. lapathifolia*, *P. minor*, and *P. bistorta* appear to be the most promising species for further studies of their antidiabetic properties using cell-based in vitro and in vivo models. The 70% aqueous ethanol extracts from *P. maculosa* and *P. hydropiper* also showed pronounced antimicrobial activity against *S. aureus*, *B. cereus*, and *P. aeruginosa*. Further experiments are required to investigate the dose–response relationships for the antibacterial and enzyme-inhibitory activities of extracts. A cluster analysis based on biochemical parameters showed that *P. hydropiper*, *P. maculosa*, and *P. lapathifolia* form a single cluster, supporting the further use of phytochemical analysis as an auxiliary tool in plant systematics.

## 1. Introduction

Interest in the study of plants of the genera *Persicaria*, *Polygonum*, and *Fallopia* is increasing due to the growing use of wild plants as components of the human diet and as medicinal raw materials [1,2]. Wild edible plants make a significant contribution to dietary diversity, providing the body with essential macronutrients, trace elements, vitamins, fiber, and antioxidants [2]. Plants of the genera *Persicaria*, *Polygonum*, and *Fallopia* are widely distributed from the northern temperate regions to tropical and subtropical zones [3] and have a long history of human use as food and medicine [4].

The effective use of plants of the genera *Persicaria*, *Polygonum*, and *Fallopia* is associated with the presence of a variety of secondary metabolites, which account for their broad range of biological activities [5]. Thus, *Persicaria hydropiper* (L.) Delarbre has been found to contain quinic acid, gallic acid, quercetin, quercetin-3-O-glucoside, quercetin-3-O-galactoside, and kaempferol-3-O-glucoside [6]. Extracts and compounds from *P. hydropiper* exhibit anti-inflammatory, antioxidant, antimicrobial, and other activities. Recent studies have demonstrated the potential of *P. hydropiper* for the treatment of neurological disorders such as Alzheimer’s disease, anxiety, and depression [7]. An extract of *Persicaria minor* Opiz, which is characterized by a high content of quercetin-3-glucuronide with antioxidant and anticholinesterase activity, may also represent a potential treatment for vascular dementia and Alzheimer’s disease [8]. Chalcones, flavanones, flavonols, diarylheptanoids, a stilbene derivative, and an α-ionol glycoside have been identified in *Persicaria maculosa* Gray [9]. Plant extracts have been reported to exhibit antifungal, antibacterial, antioxidant, and neuroprotective effects [3]. It has been shown that various extracts and isolated metabolites of *Persicaria lapathifolia* (L.) Delarbre exhibit antimicrobial, anthelmintic, antiemetic, anticancer, antioxidant, α/β-glucosidase inhibitory, and anticholinesterase activities. These properties are attributed to the numerous phytocomponents of this plant, such as flavonoids, chalcones, acylated flavonoids, ferulate esters, and phenolic acids [10]. In *Persicaria amphibia* (L.) Delarbre, glucuronide derivatives of quercetin (quercetin 3-O-[α-rhamnosyl-(1→2)-β-glucuronide] and quercetin 3-O-β-glucuronide) with anticancer activity have been identified [11]. A study on the isolation of the major phenolic compounds from *Persicaria bistorta* (L.) Samp. demonstrated the presence of rare malonylated derivatives of quercetin and kaempferol with anti-inflammatory activity [12].

The chemical composition and biological activity of *Polygonum aviculare* L. have been well studied. In traditional medicine, this plant has been used to treat respiratory and skin diseases and as a tonic, diuretic, astringent, hemostatic, and antihypertensive agent [7]. The medicinal properties of *Polygonum aviculare* L. are attributed to its phytochemical composition, particularly the presence of phenolic acids and flavonoids, especially flavonol glucuronides (e.g., quercetin 3-O-β-D-glucuronide, kaempferol 3-O-β-D-glucuronide, and isorhamnetin 3-O-β-D-glucuronide) [13,14].

The phytochemical composition and biological activity of *Fallopia* plants have received less attention in the literature. A number of studies have shown that *Fallopia convolvulus* (L.) Á. Löve contains biologically active compounds such as flavonoids (mainly quercetin 3-O-galactoside and quercetin 3-O-glucoside), anthraquinones, phenolic acids, and steroids, which exhibit antioxidant, anti-inflammatory, anticancer, and antibacterial activities [15,16].

Additional interest in plants of the genera *Persicaria*, *Polygonum*, and *Fallopia* is associated with their complex systematic classification. Previously, plants of the genera *Persicaria*, *Polygonum*, and *Fallopia* were included in a single genus, *Polygonum* (family Polygonaceae). However, due to significant morphological, phylogenetic, and phytochemical differences, they were separated into distinct genera [17]. There are several approaches to plant taxonomy [18]. The classical approach is based primarily on stable morphological features that persist regardless of environmental conditions. The second approach is based on the identification and classification of plants using molecular markers (DNA-based methods). In particular, this approach forms the basis of the APG IV (Angiosperm Phylogeny Group Classification) system [19]. Equally important today is chemotaxonomy, which is based on the classification of plants according to their chemical composition. Although the development of chemotaxonomy began quite a long time ago, modern methods of metabolomic and proteomic analysis have significantly expanded the scope of this field [20,21]. Chemotaxonomy is especially important for medicinal plants, as it contributes to the authentication of plant raw materials, standardization, and quality control [21,22]. Secondary metabolites belonging to the sesquiterpene and flavonoid groups are important markers in the chemotaxonomy of the genera *Persicaria*, *Polygonum*, and *Fallopia* [4,23,24]. The medicinal properties of plants of these genera are also largely associated with these groups of phytocompounds [3,6,17].

The purpose of this work was to conduct a comparative study of the phenolic compounds and the antioxidant, antimicrobial, and α-amylase-/α-glucosidase-inhibitory activities of extracts from *Persicaria lapathifolia* (L.) Delarbre, *Persicaria hydropiper* (L.) Delarbre, *Persicaria maculosa* Gray, *Persicaria amphibia* (L.) Delarbre, *Persicaria minor* Opiz, *Persicaria bistorta* (L.) Samp., *Polygonum aviculare* L., and *Fallopia convolvulus* (L.) Á. Löve. Although some studies have focused on the phenolic compounds and biological activities of some of these species, the novelty of this study lies in the comparison of the phenolic compound content and biological activities of these species growing under similar environmental conditions. The study will make it possible not only to assess the potential of these plants for use as food and medicinal raw materials but also to confirm the possibility of using biochemical indicators as additional tool to refine, adjust, and support existing systematic approaches.

## 2. Results

### 2.1. Total Contents of Some Groups of Phenolic Compounds

**Figure 1:**
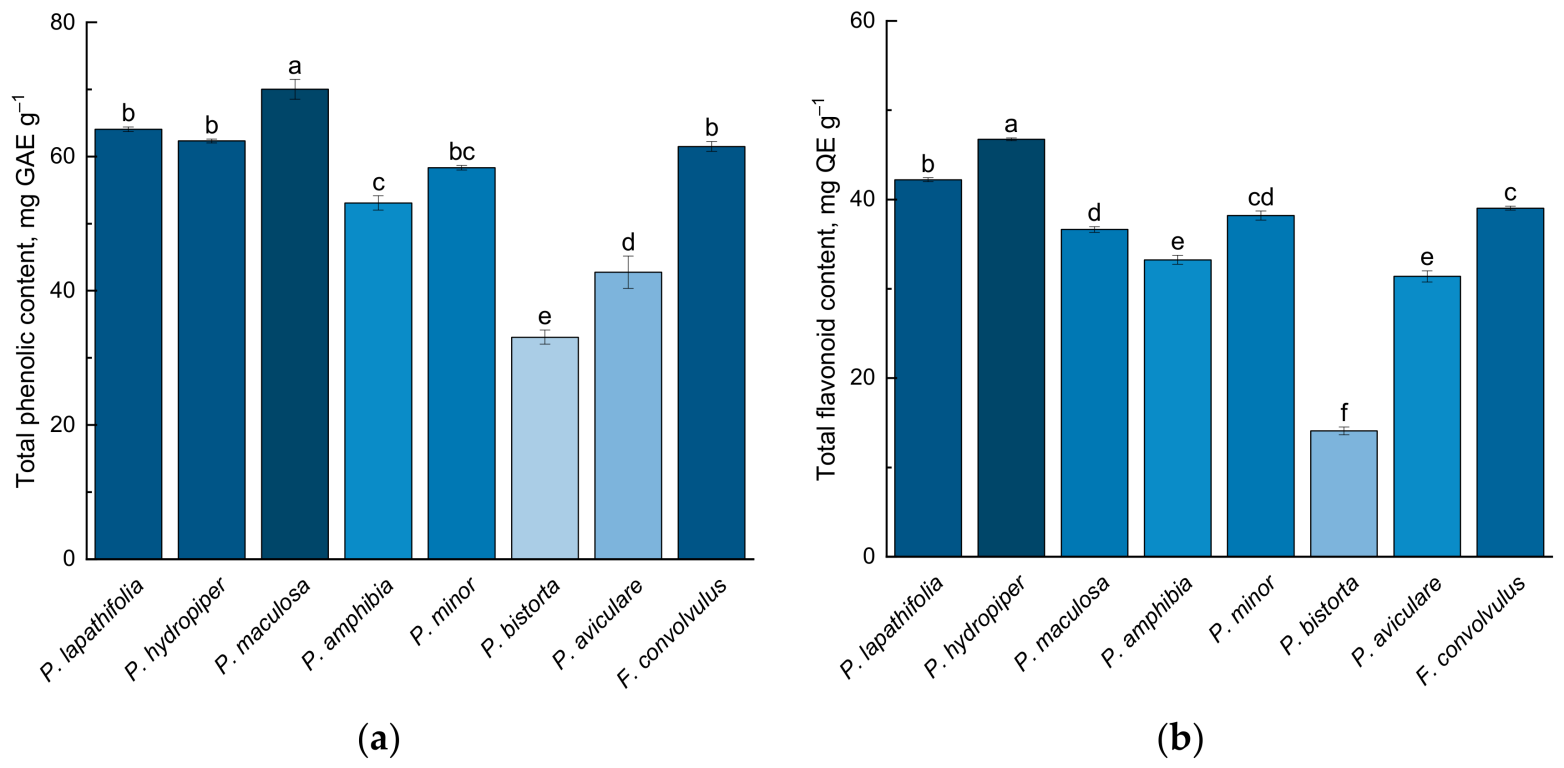

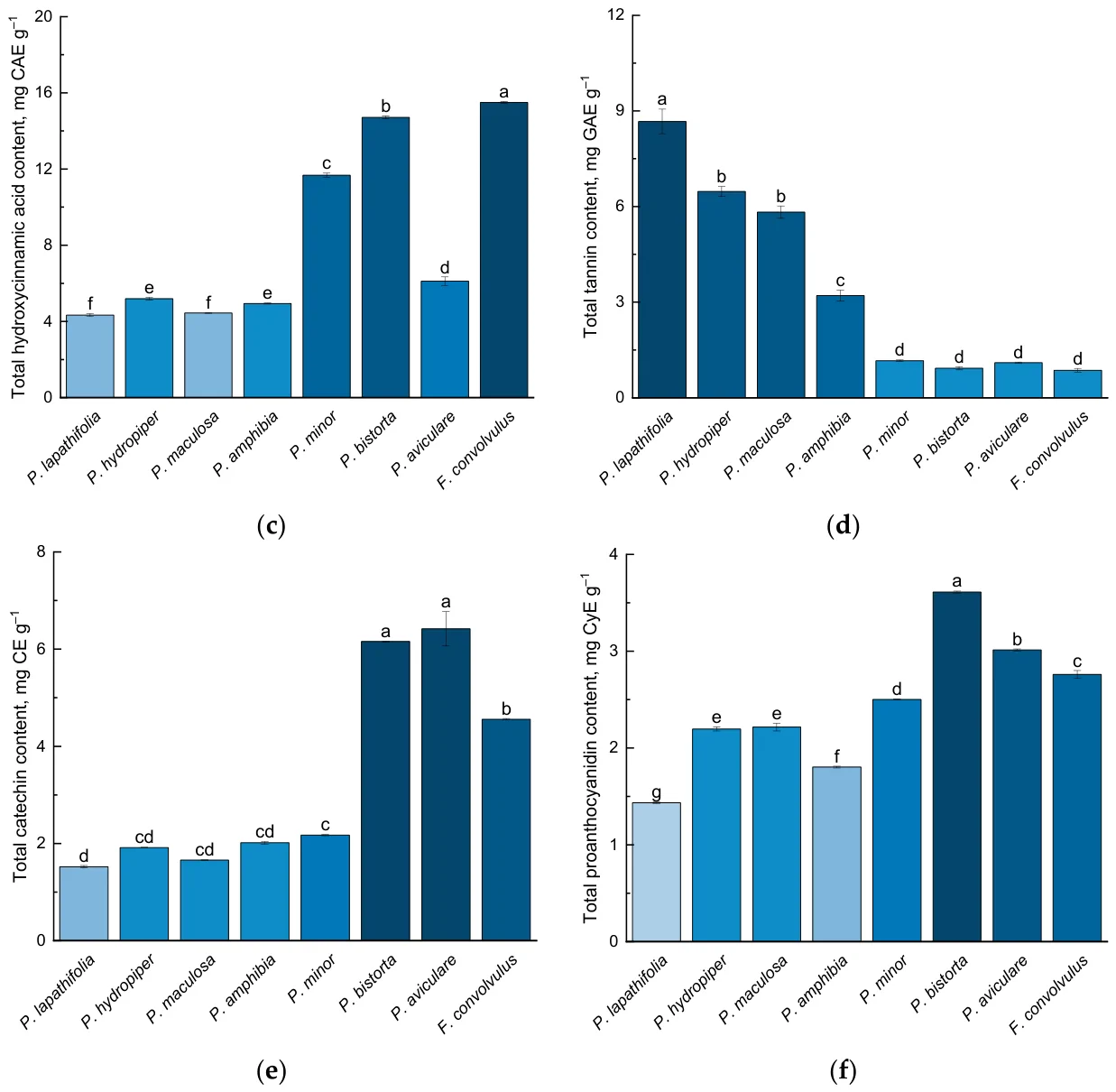
Content of some groups of phenolic compounds in different plant species of the genera *Persicaria*, *Polygonum*, and *Fallopia*: (**a**) total phenolic content; (**b**) total flavonoid content; (**c**) total hydroxycinnamic acid content; (**d**) total tannin content; (**e**) total catechin content; (**f**) total proanthocyanidin content. Different letters indicate significant differences between plant species according to Tukey’s post hoc test (*p* ≤ 0.05; *n* = 4). GAE—gallic acid equivalents; QE—quercetin equivalents; CAE—chlorogenic acid equivalents; CE—catechin equivalents; CyE—cyanidin equivalents.

The analysis of the total contents of selected groups of phenolic compounds (total phenols, flavonoids, hydroxycinnamic acids, tannins, catechins, and proanthocyanidins) showed considerable variability among the plant species studied (Figure 1).

The highest total content of phenolic compounds was observed in *P. maculosa* (about 70 mg g^−1^) (Figure 1a). High levels of phenolic compounds were also found in *P. lapathifolia*, *P. hydropiper*, and *F. convolvulus* (61.5–64.1 mg g^−1^). The lowest total content of phenolic compounds was observed in *P. bistorta* (about 33 mg g^−1^).

The highest flavonoid contents were found in *P. lapathifolia* and *P. hydropiper* and ranged from 42.2 to 46.7 mg g^−1^ (Figure 1b). The lowest flavonoid content was determined in *P. bistorta* (about 14 mg g^−1^). In all other species studied, the flavonoid content varied from 31.4 to 39.0 mg g^−1^.

In contrast to the patterns observed for total phenols, flavonoids, and tannins, high total contents of hydroxycinnamic acids were found in *P. minor*, *P. bistorta*, and *F. convolvulus* (Figure 1c). The hydroxycinnamic acid content in these species ranged from 11.7 to 15.5 mg g^−1^. In all other studied species, the content of this group of phenolic compounds was 2–3 times lower.

*P. lapathifolia* was also characterized by the highest total tannin content (about 8.7 mg g^−1^) (Figure 1d). High contents of this group of phenolic compounds were also found in *P. hydropiper* and *P. maculosa* (6.5 and 5.8 mg g^−1^, respectively). In *P. minor*, *P. bistorta*, *P. aviculare*, and *F. convolvulus*, the tannin content was significantly lower and ranged from 0.86 to 1.2 mg g^−1^.

The highest catechin levels were found in *P. bistorta* and *P. aviculare*, ranging from 6.2 to 6.4 mg g^−1^ (Figure 1e). An intermediate catechin content was determined in *F. convolvulus* (about 4.5 mg g^−1^). In all other species studied, the catechin content was 2.5–3.0 times lower, ranging from 1.5 to 2.1 mg g^−1^.

High levels of proanthocyanidins were also found in *P. bistorta*, *P. aviculare*, and *F. convolvulus* (Figure 1f). The highest content was observed in *P. bistorta* (about 3.6 mg g^−1^), whereas the lowest content of proanthocyanidins was found in *P. lapathifolia* (about 1.4 mg g^−1^).

### 2.2. Content of Individual Phenolic Compounds

The results of the high-performance liquid chromatography (HPLC) analysis showed considerable variability in the qualitative and quantitative composition of individual phenolic compounds in 70% aqueous ethanol extracts of the studied plant species (Table 1).

**Figure A1:**
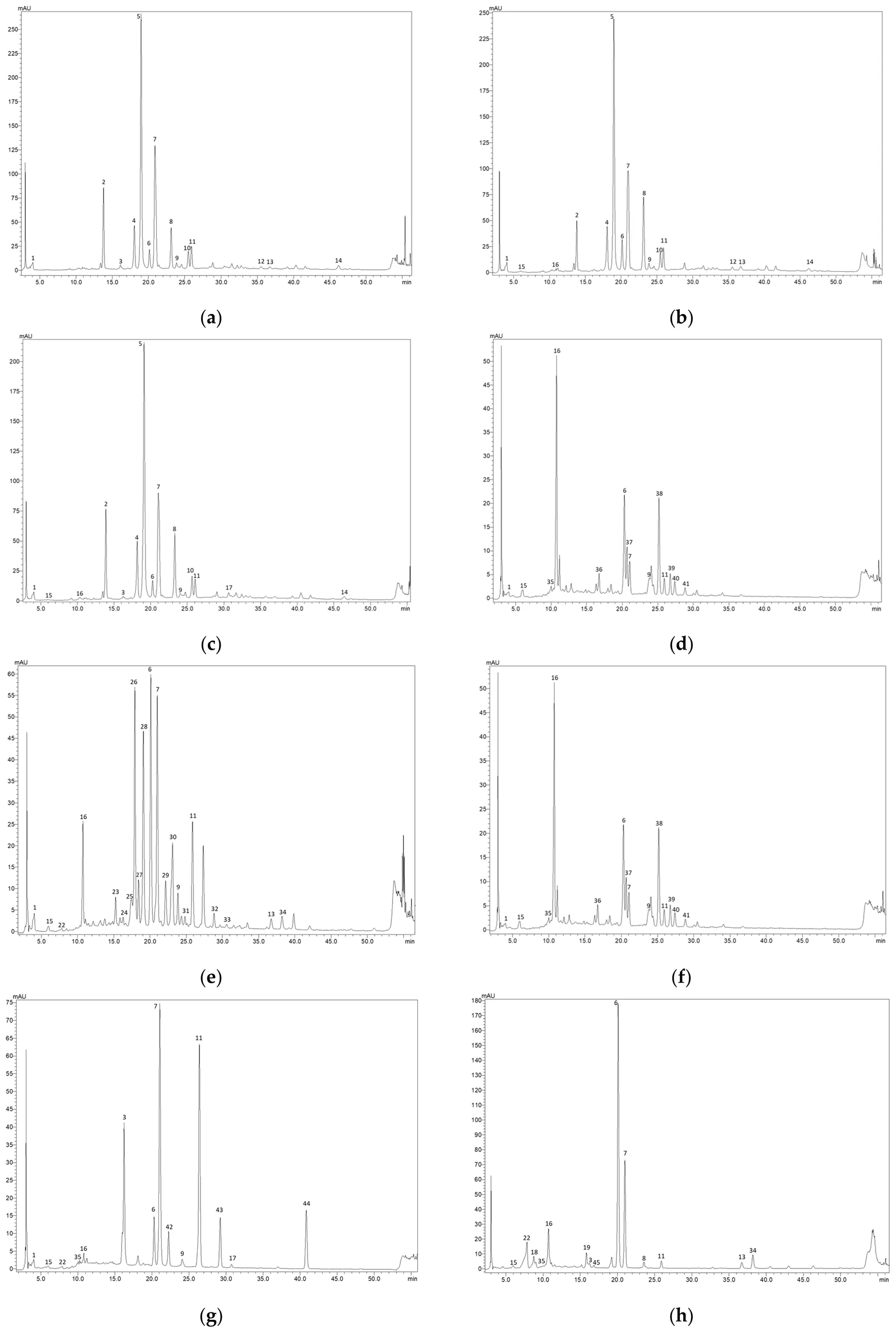
Chromatographic profile of the phenolic acids and flavonoids at 254 nm for (**a**) *Persicaria lapathifolia*, (**b**) *Persicaria hydropiper*, (**c**) *Persicaria maculosa*, (**d**) *Persicaria amphibia*, (**e**) *Persicaria minor*, (**f**) *Persicaria bistorta*, (**g**) *Polygonum aviculare*, (**h**) *Fallopia convolvulus*: 1—gallic acid (4.0); 2—ellagic acid derivative (13.8); 3—quercetin derivative (16.1); 4—ellagic acid derivative (18.0); 5—ellagic acid (19.0); 6—quercetin 3-β-D-glucoside (19.9); 7—quercetin derivative (20.9); 8—quercetin derivative (23.1); 9—kaempferol derivative (23.9); 10—quercetin derivative (25.5); 11—kaempferol 3-O-glucoside (25.9); 12—ellagic acid derivative (35.5); 13—quercetin derivative (36.7); 14—hesperetin (46.2); 15—3,4-dihydroxybenzoic acid (protocatechuic acid) (6.0); 16—caffeic acid (9.0); 17—kaempferol derivative (30.6); 18—caftaric acid (10.8); 19—quercetin derivative (15.8); 20—quercetin derivative (16.1); 21—ellagic acid derivative (28.1); 22—chlorogenic acid (7.9); 23—scutellarein derivative (15.3); 24—luteolin derivative (16.3); 25—scutellarein derivative (17.3); 26—quercetin derivative (17.9); 27—luteolin derivative (18.4); 28—scutellarein derivative (19.1); 29—apigenin derivative (22.2); 30—apigenin derivative (22.9); 31—luteolin derivative (24.8); 32—quercetin derivative (27.3); 33—luteolin derivative (30.6); 34—luteolin derivative (38.2); 35—catechin (10.0); 36—luteolin derivative (16.7); 37—apigenin derivative (20.7); 38—kaempferol derivative (25.2); 39—quercetin derivative (26.8); 40—kaempferol derivative (27.4); 41—quercetin derivative (28.8); 42—kaempferol derivative (22.4); 43—morin; 44—kaempferol derivative (40.8); 45—ferulic acid (16.8).

Among the phenolic acids identified in the studied species were gallic acid, 3,4-dihydroxybenzoic acid, chlorogenic acid, caftaric acid, caffeic acid, ferulic acid, ellagic acid, and its derivatives (Table 1, Appendix A, Figure A1). While gallic, 3,4-dihydroxybenzoic, and caffeic acids were found in almost all studied species, the remaining acids showed high species specificity. Thus, chlorogenic acid was found only in *P. minor*, *P. aviculare*, and *F. convolvulus*; caftaric acid was found in *P. amphibia* and *F. convolvulus*; and ferulic acid was found only in *F. convolvulus*. It should be noted that ellagic acid and its derivatives were characteristic only of *P. lapathifolia*, *P. hydropiper*, *P. maculosa*, and *P. amphibia*. At the same time, the content of ellagic acid and its derivatives in *P. lapathifolia*, *P. hydropiper*, and *P. maculosa* was high (on average, about 15 mg g^−1^) and significantly exceeded the levels of the other phenolic acids.

Among the flavonoids identified in the studied species were catechin, quercetin and its derivatives, kaempferol derivatives, morin, hesperetin, luteolin derivatives, apigenin derivatives, and scutellarein derivatives (Table 1, Appendix A, Figure A1). Flavonols (derivatives of quercetin and kaempferol) were more characteristic of the studied species than flavones (derivatives of luteolin and apigenin). Thus, quercetin 3-β-D-glucoside and kaempferol 3-O-glucoside were identified in all studied species. However, the concentrations of these compounds varied considerably among species. Higher contents of quercetin derivatives were characteristic of *P. hydropiper*, *P. amphibia*, and *F. convolvulus* (on average, about 13 mg g^−1^), whereas a higher content of kaempferol derivatives was characteristic of *P. aviculare* (about 7.8 mg g^−1^). Catechin was found only in *P. bistorta*, *P. aviculare*, and *F. convolvulus*; morin only in *F. convolvulus*; hesperetin in *P. lapathifolia*, *P. hydropiper*, and *P. maculosa*; luteolin derivatives in *P. minor*, *P. bistorta*, and *F. convolvulus*; and apigenin derivatives in *P. minor* and *P. bistorta*. Scutellarein derivatives were identified in *P. minor*, where their content was relatively high, at about 5.1 mg g^−1^.

### 2.3. Antioxidant Activity of the Plant Extracts

The highest antioxidant activity, measured by 2,2-diphenyl-1-picrylhydrazyl (DPPH), 2,2′-azino-bis(3-ethylbenzothiazoline-6-sulfonic acid) (ABTS), and ferric reducing antioxidant power (FRAP) assays (Table 2), was exhibited by *P. maculosa* extracts (about 337, 211, and 191 mg Trolox equivalents (TE) g^−1^, respectively). High levels of antioxidant activity were also characteristic of extracts of *P. lapathifolia*, *P. hydropiper*, and *F. convolvulus*. The lowest antioxidant activity was observed for *P. aviculare* extracts (about 130, 121, and 64 mg TE g^−1^, respectively).

### 2.4. Inhibitory Activity of the Plant Extracts Against α-Amylase and α-Glucosidase

**Figure 2:**
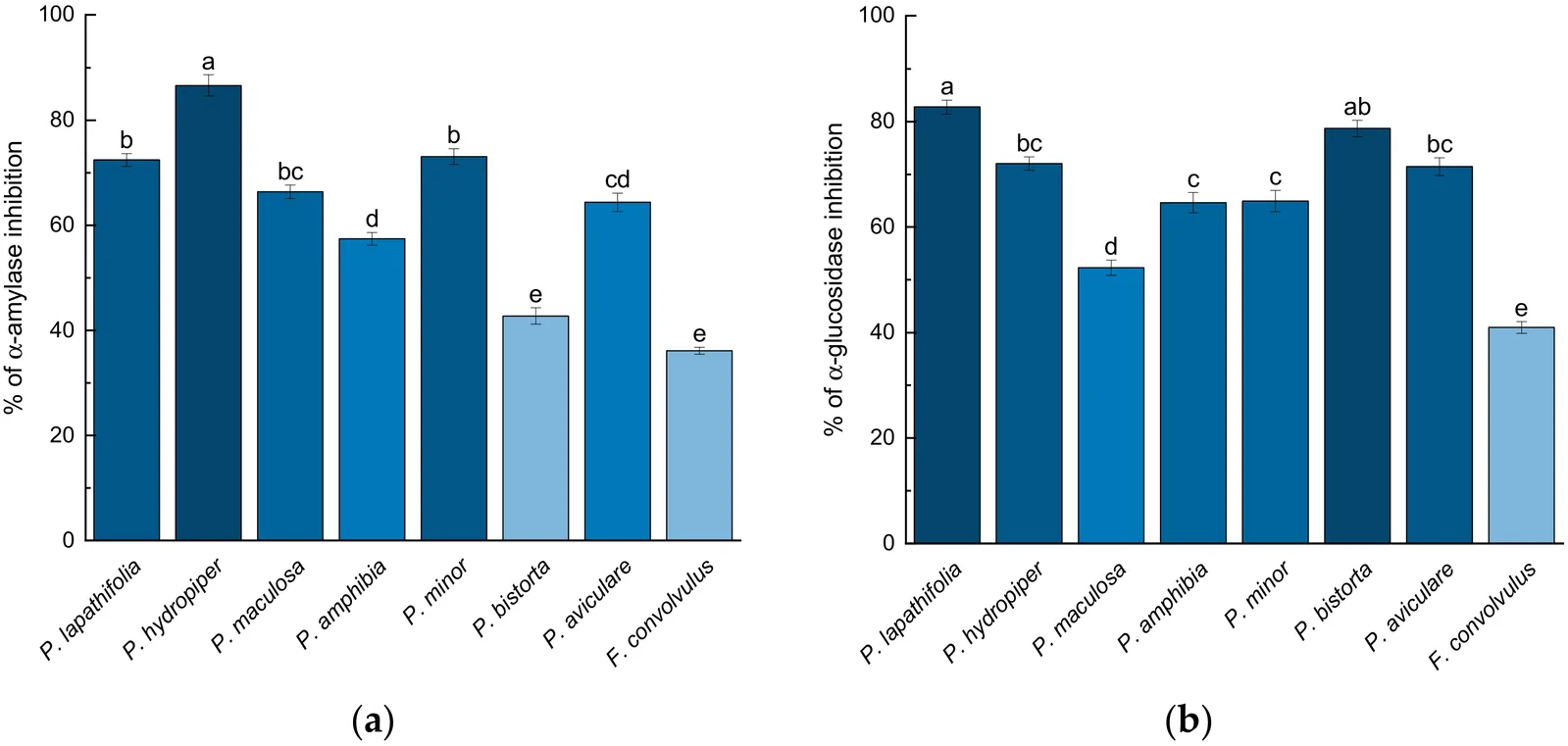
α-Amylase and α-glucosidase inhibitory activities of extracts (2 mg mL^−1^) from plants of the genera *Persicaria*, *Polygonum,* and *Fallopia*: (**a**) α-Amylase inhibitory activity; (**b**) α-Glucosidase inhibitory activity. Different letters indicate significant differences between plant species according to Tukey’s post hoc test (*p* ≤ 0.05; *n* = 4). Acarbose (0.1 mg mL^−1^) was used as the reference standard: α-amylase inhibition—79 ± 3%; α-glucosidase inhibition—96 ± 6%.

The in vitro inhibitory activity of plant extracts against α-amylase and α-glucosidase was evaluated by measuring the percentage inhibition of each enzyme, with acarbose as the positive control. The results are shown in Figure 2.

Inhibitory activity against α-amylase was observed for all studied species, but the degree of inhibition varied considerably among species (Figure 2a). The strongest inhibitory effect was found for *P. hydropiper* extracts (about 86%), followed by *P. lapathifolia* and *P. minor* extracts (72–73%).

Extracts of all studied plant species also showed inhibitory activity against α-glucosidase (Figure 2b). The highest inhibitory activity was observed for extracts of *P. lapathifolia* and *P. bistorta* (83% and 79%, respectively).

### 2.5. Antimicrobial Activity of the Plant Extracts

Analysis of antimicrobial activity showed that 70% aqueous ethanol extracts of the studied plants exhibited antibacterial activity against the Gram-positive bacteria (*S. aureus* and *B. cereus*) and the Gram-negative bacterium *P. aeruginosa* but did not exhibit antifungal activity (Table 3). The extracts of *P. maculosa* and *P. hydropiper* showed the highest antimicrobial activity against *S. aureus*, whereas the *P. hydropiper* extract showed the highest activity against *B. cereus* and *P. aeruginosa*.

### 2.6. Correlation and Cluster Analysis

**Figure 3:**
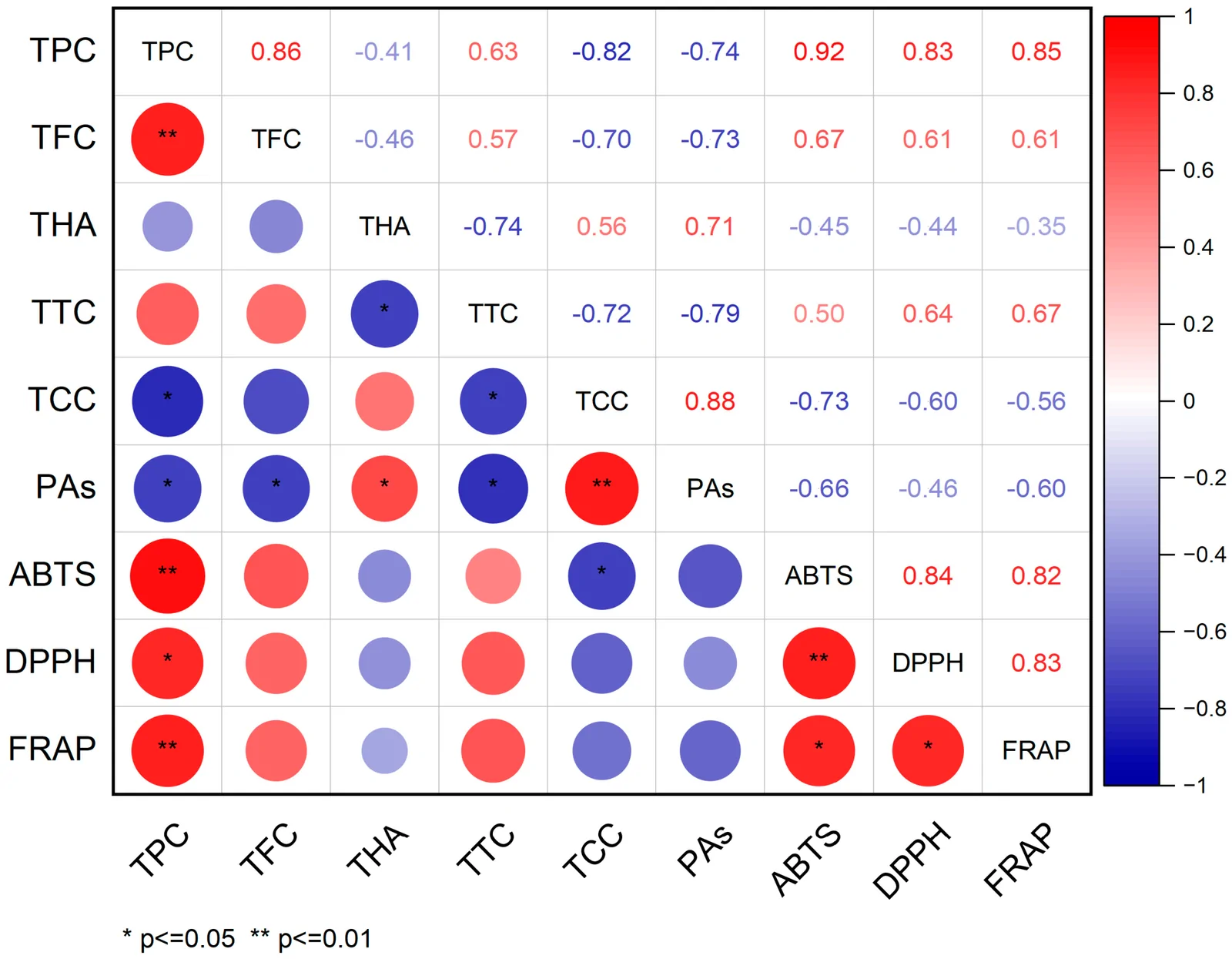
Pearson correlation coefficients between phenolic compounds and antioxidant activity of plant extracts. TPC—total phenolic content; TFC—total flavonoid content; THA—total hydroxycinnamic acids; TTC—total tannin content; TCC—total catechin content; Pas—total proanthocyanidin content; ABTS—antioxidant activity determined by the ABTS (2,2′-azino-bis(3-ethylbenzothiazoline-6-sulfonic acid)) assay; DPPH—antioxidant activity determined by the DPPH (2,2-diphenyl-1-picrylhydrazyl) assay; FRAP—ferric reducing antioxidant power.

To identify the relationships between the contents of individual groups of phenolic compounds and the antioxidant activity of the extracts, a correlation matrix was constructed based on Pearson correlation coefficients (Figure 3). A significant correlation was found between the total phenolic content and antioxidant activity (ABTS: r = 0.92, *p* ≤ 0.01; FRAP: r = 0.85, *p* ≤ 0.01; DPPH: r = 0.83, *p* ≤ 0.05). The results for the correlation between catechin content and antioxidant activity were unexpected. A negative relationship between these parameters was observed. For antioxidant activity measured by the ABTS method, this negative correlation was statistically significant (r = −0.73, *p* ≤ 0.05).

**Figure 4:**
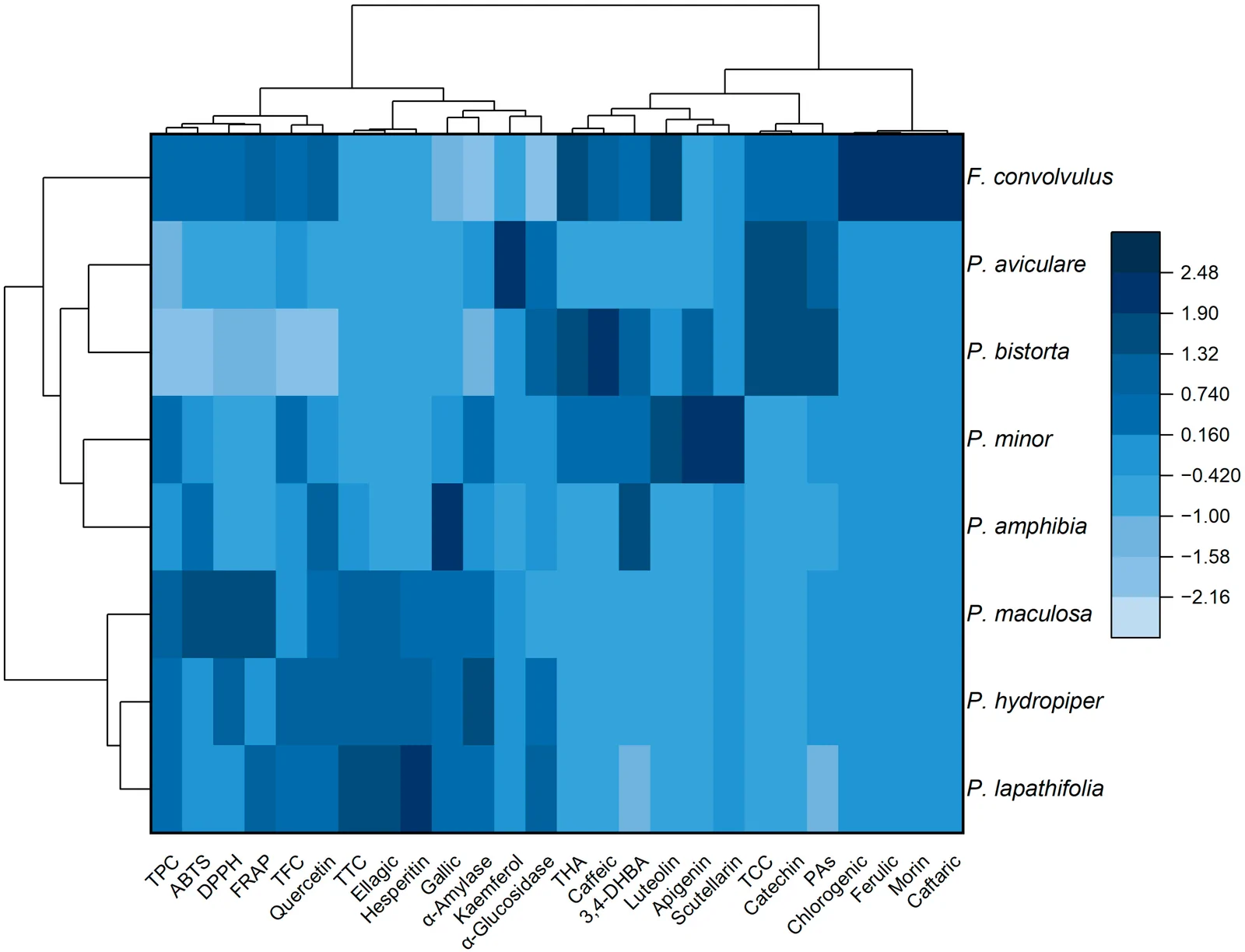
Heat map with clusters of the studied phytochemical parameters (at the top) and plant species (on the left). TPC—total phenolic content; TFC—total flavonoid content; THA—total hydroxycinnamic acids; TTC—total tannin content; TCC—total catechin content; Pas—total proanthocyanidin content; ABTS—antioxidant activity determined by the ABTS (2,2′-azino-bis(3-ethylbenzothiazoline-6-sulfonic acid)) assay; DPPH—antioxidant activity determined by the DPPH (2,2-diphenyl-1-picrylhydrazyl) assay; FRAP—ferric reducing antioxidant power; 3,4-DHBA—3,4-dihydroxybenzoic acid.

Based on the normalized values of the contents of individual groups of phenolic compounds and individual phenolic compounds, antioxidant activity, and inhibitory activity against glycolytic enzymes, cluster analysis was performed and a heat map was constructed (Figure 4). According to the cluster analysis, the studied plant species were divided into two major clusters. The first cluster included representatives of the genus *Persicaria*: *P. lapathifolia*, *P. hydropiper*, and *P. maculosa*. Three subclusters could be distinguished within the second cluster. The first included species of the genus *Persicaria*, *P. minor* and *P. amphibia*; the second included *Persicaria bistorta* and *Polygonum aviculare*; and the third included *Fallopia convolvulus*.

## 3. Discussion

### 3.1. Phenolic Compounds

Plants of the genera *Persicaria*, *Polygonum*, and *Fallopia* are characterized by a diverse phytochemical composition. Among these compounds, sesquiterpenes and phenolic compounds are particularly important classes characteristic of these plants. Our study focused on the quantitative contents of individual groups of phenolic compounds and selected phenolic acids and flavonoids. The content of phenolic compounds in plants can be influenced by various factors, including geographical origin, plant maturity, and environmental conditions [25,26,27]. To ensure a valid comparison of the studied species in terms of the content and composition of phenolic compounds, samples of all species were collected from experimental plots located no more than 50 m apart, thereby ensuring identical geographical origin and environmental conditions. Moreover, all experimental plants were harvested during the flowering phase. It should be noted, however, that while the experimental design permits comparison among species, the results and conclusions presented here are restricted to a single population per species. Generalization to the taxon as a whole would therefore require a broader sampling design.

The extraction solvent and technique are other critical factors influencing the content and composition of phenolic compounds and, consequently, the biological activity of the extracts [21]. Based on previously published studies [28,29,30,31], 70% aqueous ethanol and ultrasound-assisted extraction were used in our study to prepare of the extracts, as these are the most effective and widely used solvent and technique, respectively.

The total content of phenolic compounds in the studied species varied from 33 mg g^−1^ in *P. bistorta* to 70 mg g^−1^ in *P. maculosa*. Species such as *P. maculosa*, *P. lapathifolia*, *P. hydropiper*, and *F. convolvulus* were distinguished by their high phenolic content. The results obtained are generally comparable with the data available in the literature. Thus, previous studies reported values of 75–90 mg g^−1^ in *P. hydropiper*, depending on growing conditions [32], and about 50 mg g^−1^ in *P. aviculare* [33]. Data have also been reported for *P. bistorta* [34]. It is known that the accumulation of phenolic compounds is strongly influenced not only by plant genotype but also by growing conditions, including light, temperature, and soil properties [35]; therefore, direct comparison of the results obtained with those available in the literature is difficult. Previously, our group studied seven species of the genus *Rumex*, belonging to the same family, Polygonaceae, and growing under similar environmental and climatic conditions [36]. It was found that the total phenolic content in these species varied from 23 to 131 mg g^−1^, i.e., it was either comparable to that of plants of the genus *Persicaria* or exceeded it by up to 1.8-fold.

Flavonoid accumulation correlated with the total content of phenolic compounds. The maximum flavonoid content was about 45 mg g^−1^ in *P. hydropiper*, which, however, was 1.5 times lower than the value reported in the study [32]. In *P. bistorta*, the total flavonoid content was the lowest (14.1 ± 0.7 mg g^−1^) and was comparable to that reported for *P. bistorta* from Northern Iran (14.7 ± 0.4 mg g^−1^) [17]. According to the HPLC results, the studied species were characterized by greater accumulation of flavonols, primarily derivatives of quercetin and kaempferol, than of flavones, including derivatives of luteolin and apigenin. A higher quantitative content and greater structural diversity of flavonol derivatives (quercetin and kaempferol) in plants of the genera *Persicaria*, *Polygonum*, and *Fallopia* have also been reported in previous studies [7,8,9,10,11,12,13,14,15,16].

The results for the total content of hydroxycinnamic acids differed from those observed for total phenolic compounds and flavonoids. *P. minor*, *P. bistorta*, and *F. convolvulus* accumulated 2–3 times more compounds of this group than the other studied species. Structurally, phenolic acids and their derivatives are divided into two major groups: hydroxybenzoic acids and hydroxycinnamic acids. The main hydroxycinnamic acids found in plants include caffeic, ferulic, sinapic, and chlorogenic acids [37,38]. According to the HPLC results, caffeic, chlorogenic, and caftaric acids accumulated in *P. minor*, *P. bistorta*, and *F. convolvulus*, distinguishing these species from the other studied plants.

The analysis of the total contents of catechins, tannins, and proanthocyanidins showed a relationship among their accumulation patterns. Thus, *P. bistorta*, *P. aviculare*, and *F. convolvulus* were characterized by high contents of catechins and proanthocyanidins (condensed tannins) and low contents of hydrolysable tannins. The opposite pattern was observed for *P. lapathifolia*, *P. hydropiper*, *P. maculosa*, and *P. amphibia*, which showed low contents of catechins and proanthocyanidins and high tannin contents. In the modern classification of plant secondary metabolites, the term “tannins” includes two groups of compounds: condensed tannins (proanthocyanidins) and hydrolysable tannins (gallotannins and ellagitannins), whose biosynthetic pathways differ [39]. Catechins and epicatechins are precursors (monomeric units) of proanthocyanidins, whereas gallic and ellagic acids are structural components of hydrolysable tannins [39,40]. The data obtained in our study, namely the high contents of catechins and proanthocyanidins in *P. bistorta*, *P. aviculare*, and *F. convolvulus* and the high contents of tannins, ellagic acid, and its derivatives in *P. lapathifolia*, *P. hydropiper*, *P. maculosa*, and *P. amphibia*, suggest that the first three species may have a more active flavan-3-ol biosynthetic pathway, whereas the latter may be characterized by a more active gallic acid biosynthetic pathway followed by its di- and/or oligomerization [41]. It should be noted, however, that a more accurate characterization of the qualitative composition of phenolic compounds in the studied species will require additional studies using solvents of different polarities, as well as high-resolution mass spectrometry (HRMS) and nuclear magnetic resonance (NMR) methods.

### 3.2. Biological Activity

The biological activity of plant extracts is determined by the presence of various phytocomponents. Phenolic compounds constitute a large group of plant secondary metabolites, many of which have demonstrated antioxidant activity due to the presence of free hydroxyl groups in their structures [42]. In this study, a significant positive correlation was found between the total content of phenolic compounds and antioxidant activity measured by the DPPH, ABTS, and FRAP methods. Although no significant positive correlation was established between antioxidant activity and the contents of the other studied groups of phenolic compounds, the analysis of individual phenolic compounds revealed several patterns. Thus, the highest antioxidant activity was observed in extracts of *P. maculosa*, *P. lapathifolia*, and *P. hydropiper*. These plants were characterized by the accumulation of ellagic acid and its derivatives. Previous studies have shown that ellagic acid exhibits pronounced antioxidant activity in various in vitro assays, including DPPH, ABTS, and FRAP [43,44]. In some assays, the antioxidant activity of ellagic acid exceeded that of such well-known antioxidants as butylated hydroxyanisole (BHA), butylated hydroxytoluene (BHT), tocopherol, and ascorbic acid [43]. *Fallopia convolvulus* extracts also exhibited high antioxidant activity and, in addition to a high content of isoquercitrin, contained higher levels of chlorogenic and caftaric acids than the other studied species. The antioxidant properties of coffee [45] and prunes [46] have also been associated with the presence of chlorogenic acid and its isomers, including neochlorogenic and cryptochlorogenic acids. Caftaric acid has also been shown to exhibit greater activity in neutralizing HOO˙ radicals than such well-known antioxidants as Trolox, ascorbic acid, and resveratrol [47]. Methods for determining antioxidant activity are known to be based on different mechanisms of antioxidant action. The FRAP method is evaluated based on the single-electron transfer (SET) mechanism, whereas the DPPH and ABTS methods involved both the hydrogen atom transfer (HAT) and SET mechanisms [48]. It has been shown that, depending on the reaction conditions, ellagic acid may exhibit three mechanisms of antioxidant action—namely, hydrogen atom transfer (HAT), single electron transfer followed by proton transfer (SET-PT), and sequential proton loss electron transfer (SPLET)—with HAT predominating in polar solvents [49]. A study of the reaction of isoquercetin with the superoxide anion radical also demonstrated that the overall antioxidant effect of this compound may involve several pathways, including Fe^2+^ binding, H-donation, electron transfer (ET), proton transfer, and even radical adduct formation [50]. In studies on the antioxidant mechanisms of chlorogenic acid and its isomers, it has been suggested that SET-PT is not the preferred antioxidant mechanism, as the formation of radical cations is a highly energy-demanding process, even in polar solvents. The authors proposed that HAT is the primary reaction pathway in nonpolar solvents, whereas in polar media, HAT and SPLET act as competing pathways [51]. Nevertheless, it should be noted that the present study examined the antioxidant activity of crude extracts containing a complex mixture of components. Mixtures of various phenolic compounds are known to exhibit synergistic, additive and antagonistic effects [52], which necessitates the isolation and purification of individual compounds, as well as further investigation of their mechanisms of action.

The inhibitory activity against α-amylase and/or α-glucosidase was demonstrated for extracts of *P. hydropiper*, *P. lapathifolia*, *P. minor*, and *P. bistorta*. Previously, α-glucosidase inhibitory activity was reported for extracts of *P. aviculare* [53] and *P. lapathifolia* [54]. For *P. hydropiper* extracts, inhibitory activity against both α-amylase and α-glucosidase has also been reported [55,56]. Using molecular docking methods, the authors identified compounds in *P. hydropiper* extracts with the highest affinity for these enzymes. Thus, kaempferol-3-(p-coumaroyl-diglucoside)-7-glucoside showed the lowest binding energy with α-glucosidase, whereas cyanidin-3-rutinoside showed the lowest binding energy with α-amylase [56]. Several studies have shown that luteolin is a more potent inhibitor of α-amylase than quercetin and its derivatives. These studies indicated that the presence of -OH groups together at positions 3 and 4 of the B-ring and at positions 5 and 7 of the A-ring increases the inhibition of α-amylase by flavonoids. A comparison of the activities of isoquercitrin and quercetin revealed that isoquercitrin is more active, indicating that substituting the hydroxyl group at the 3-position of the C-ring facilitates more effective binding to α-amylase compared with that of quercetin [57]. Conversely, studies on the α-glucosidase inhibitory activity of various flavonoids have shown that, as a rule, glycosylation, particularly at the 3-position of the C-ring, leads to reduced inhibitory activity [57,58]. Several hypotheses have been proposed to explain this phenomenon, including increased steric hindrance, increased molecular size and polarity, and the presence of a non-planar structure—factors that can influence the binding affinity of flavonoids [58]. A comparative study of the inhibitory activity of flavonoids against α-amylase and α-glucosidase revealed that, for α-amylase inhibition, the flavonoid structure must contain a double bond between carbon atoms C2 and C3, as well as hydroxyl groups at position 5 of the A-ring and at positions 3 and 4 of the B-ring. For α-glucosidase inhibition, the structure must contain hydroxyl groups at positions 3 and 4 of the B-ring and at position 3 of the C-ring [59]. It must be highlighted that in present study, a yeast-derived α-glucosidase was used. Because mammalian-derived α-glucosidase is difficult to obtain, while yeast-derived α-glucosidase is commercially available, offering a more cost-effective and accessible alternative. Therefore, it is widely employed in preliminary screening and in vitro studies [60]. However, due to several differences in size and amino acid sequences among yeast, rat, and human intestinal α-glucosidases, additional research linking rat and human intestinal enzymes to cell-based experiments is necessary to confirm the antidiabetic activity of the extract [61]. Furthermore, the percentage enzyme inhibition values obtained in this study should be regarded as a semi-quantitative assessment of the inhibitory activity of the extracts. Consequently, the most promising extracts (*P. hydropiper*, *P. lapathifolia*, *P. minor*, and *P. bistorta*) should be evaluated against human α-amylase and α-glucosidase to determine IC_50_ values, and their antidiabetic potential should be further explored in both in vitro and in vivo models.

Preliminary screening of the antibacterial activity of plant extracts was performed using an agar diffusion assay. The most pronounced antibacterial activity was exhibited by extracts of *P. hydropiper* and *P. maculosa*, which were characterized by high contents of ellagic acid and quercetin derivatives. Studies on the antibacterial mechanisms of ellagic acid have shown that it disrupts bacterial morphology, damages cell membranes and cell walls, inhibits biofilm formation, and suppresses bacterial respiratory metabolism [62]. Flavonoids can inhibit bacterial nucleic acid synthesis, energy metabolism, biofilm formation, and cytoplasmic membrane integrity; hydroxyl groups enhance antibacterial activity, whereas hydrophobic substituents facilitate flavonoid penetration across the bacterial membrane [63]. The structural features of flavonoids play an important role in their antibacterial activity. Thus, it has been shown that hydroxylation at positions 5 and 7 of the A-ring, hydroxylation at positions 3 and 4 of the B-ring, and geranylation or prenylation at position 6 of the A-ring determine their antibacterial activity. However, a key condition is that the flavonoids retain their amphiphilic properties, enabling them to penetrate bacterial cells and exert a pronounced antibacterial effect [64]. In general, the antimicrobial properties of various plants of the genera *Persicaria* and *Polygonum* have been well studied [17]. However, it should be noted that, in contrast to previous reports [65,66,67,68], no antifungal activity against *C. albicans* was detected in the present study. This discrepancy may be due to several factors, including differences in the plant parts used for analysis and in the solvents used for extraction. For example, polygodial, a sesquiterpene dialdehyde with pronounced antifungal activity, is extracted more efficiently with dichloromethane (DCM) [23]. The present study, by contrast, focused primarily on phenolic compounds, and extraction was performed using 70% aqueous ethanol. Although flavonoids and phenolic acids are known to exhibit antimicrobial properties, the presence and content of compounds from other classes, such as terpenoids, including mono- and sesquiterpenes, should also be taken into account. Furthermore, it is necessary to consider the primary limitation of the agar diffusion method—namely, the varying diffusion rates of different compounds and the consequent need for caution when comparing the sizes of inhibition zones for extracts of differing composition [69]. Nevertheless, this method remains a useful tool for the rapid and simple preliminary qualitative screening of the antimicrobial activity of plant extracts, essential oils, and natural compounds [69,70,71].

### 3.3. Biochemical Marker Clustering in Plant Systematics

According to recent molecular studies based on analyses of the plastid genomes of various species of the genera *Persicaria*, *Polygonum*, and *Fallopia*, the most closely related species are *P. hydropiper*, *P. maculosa*, and *P. lapathifolia*, which are included in section *Persicaria* [72,73,74]. The results of the cluster analysis obtained in our study confirm the close relationship among these plant species in terms of their biochemical characteristics. According to molecular genetic studies, *P. amphibia* is placed in the separate section *Amphibia* [72,73,74]. In our study, this species was grouped in the same cluster as *P. minor*. Unfortunately, no data were found in the literature or databases regarding the phylogenetic position of the latter species among other *Persicaria* species. According to our analysis, *P. bistorta* and *P. aviculare* also formed a separate cluster, the most distant from section *Persicaria*. According to molecular studies, *Polygonum aviculare* is indeed phylogenetically distant from the genus *Persicaria* [73]. The placement of *F. convolvulus* in a separate cluster based on biochemical parameters is also consistent with its assignment to the separate genus *Fallopia* [75]. Thus, cluster analysis based on the contents of phenolic compounds and the biological activities of extracts of *Persicaria lapathifolia*, *Persicaria hydropiper*, *Persicaria maculosa*, *Persicaria amphibia*, *Persicaria minor*, *Persicaria bistorta*, *Polygonum aviculare*, and *Fallopia convolvulus* demonstrated the potential of approaches based on biochemical markers as a viable supplementary tool to the existing systematic classification of species within the genus *Persicaria* and related genera.

## 4. Materials and Methods

### 4.1. Plant Material and Preparation of Extracts

The aboveground parts of *Persicaria lapathifolia* (L.) Delarbre, *Persicaria hydropiper* (L.) Delarbre, *Persicaria maculosa* Gray, *Persicaria amphibia* (L.) Delarbre, *Persicaria minor* Opiz, *Persicaria bistorta* (L.) Samp., *Polygonum aviculare* L., and *Fallopia convolvulus* (L.) Á. Löve were harvested during the flowering phase in July–August 2022. The collection was carried out in the vicinity of the village of Otradnoye (Kaliningrad Region, Russia), away from roads and agricultural fields. Plant material was collected from four experimental sites located 20–50 m apart. At each site, 2–5 plants were collected and combined into one sample. Plant material from each of the four experimental sites represented a biological replicate, and was analyzed separately (*n* = 4). The species were identified by A. Pungin, PhD. The taxonomy and scientific names of the species were obtained from the World Flora Online (WFO) database (https://www.worldfloraonline.org/). Voucher specimens were deposited in the Herbarium of Immanuel Kant Baltic Federal University (KLGU Herbarium; vouchers: *P. lapathifolia* KLGU001467; *P. hydropiper* KLGU001424; *P. maculosa* KLGU001468; *P. amphibia* KLGU001452; *P. minor* KLGU001439; *P. bistorta* KLGU001461; *P. aviculare* KLGU001298; *F. convolvulus* KLGU001512).

The plant material was dried at 40 °C, ground, and sieved through a sieve with a mesh size of 2–3 mm. Phenolic compounds were extracted with 70% aqueous ethanol. Samples of plant material (0.5 g) were placed in a round-bottom flask, 20 mL of 70% EtOH was added, and the flask was placed in an ultrasonic bath for 30 min (100 W, 35 kHz). The extraction was repeated twice. The extracts were transferred to a 50 mL volumetric flask and brought to volume with 70% ethanol.

### 4.2. Determination of the Phenolic Compounds

In the extracts prepared as described above, the total contents of phenolic compounds, flavonoids, hydroxycinnamic acids, catechins, tannins, and proanthocyanidins were determined.

The total content of phenolic compounds was determined spectrophotometrically using the Folin–Ciocalteu reagent [76] and was expressed as mg of gallic acid equivalents per gram of dry weight. The total content of flavonoids was determined based on the formation of a complex between flavonoids and aluminum chloride in the presence of sodium acetate [77] and was expressed as mg of quercetin equivalents per gram of dry weight. The total content of hydroxycinnamic acids was determined using the Arno reagent [78] and was expressed as mg of chlorogenic acid equivalents per gram of dry weight. Spectrophotometric measurements for the determination of the total contents of phenolic compounds, flavonoids, and hydroxycinnamic acids were performed using a CLARIOstar microplate reader (BMG Labtech, Ortenberg, Germany).

The catechin content was determined spectrophotometrically with the vanillin reagent [79] and was expressed as mg of catechin equivalents per gram of dry weight. The total content of tannins was determined using the Prussian Blue method [80] with gallic acid used as the standard. The total tannin content was expressed as mg of gallic acid equivalents per gram of dry weight. The proanthocyanidin content was determined spectrophotometrically as described in [81] and was expressed as mg of cyanidin equivalents per gram of dry weight. Spectrophotometric measurements for the determination of the total contents of tannins, catechins, and proanthocyanidins were performed using a UV-3600 spectrophotometer (Shimadzu, Kyoto, Japan).

Chromatographic measurements were conducted using a Shimadzu LC-20 Prominence system (Shimadzu, Kyoto, Japan) equipped with an SPD20MA diode-array detector. The extracts prepared according to Section 2.1 were passed through a 0.22 µm cellulose acetate membrane before analysis. Analyte separation was achieved on a Phenomenex Luna C18 column (250 × 4.6 mm; particle size, 5 µm; Phenomenex, Torrance, CA, USA), maintained at 40 °C. The injection volume was 10 µL, and the mobile phase was delivered at a flow rate of 0.85 mL min^−1^. The mobile phase consisted of water containing trifluoroacetic acid (99.9:0.1, *v*/*v*; eluent A) and acetonitrile (eluent B). The gradient was defined by the following time points and corresponding proportions of eluent B: 5% at 0 min, 12% at 3 min, 25% at 46 min, 90% at 49.5 and 52 min, and 5% at 52.7 and 59 min. Eluent A accounted for the remaining proportion at each time point. Detector data were acquired over the wavelength range of 180–750 nm, and the resulting chromatograms were processed in LabSolutions software (LC Solution version 1.25SP1 Single PDA, Shimadzu, Kyoto, Japan). Compound identities were established by matching both retention times and UV spectra with those of chromatographically pure reference standards. Phenolic compound concentrations were determined using calibration curves covering the range of 10–100 µg mL^−1^.

Calibration standards were prepared as solutions of caftaric, chicoric, chlorogenic, neochlorogenic, p-coumaric, rosmarinic, sinapic, trans-caffeic, ferulic, 3,4-dihydroxybenzoic, gallic, and ellagic acids, as well as luteolin 7-O-glucoside, apigenin 7-O-glucoside, apigenin 7-O-glucuronide, quercetin 3-O-rutinoside, quercetin 3-β-D-glucoside, kaempferol 3-O-glucoside, hesperetin, baicalin, scutellarein, diosmin, morin, and catechin. All standards were purchased from Sigma-Aldrich (Sigma-Aldrich Rus, Moscow, Russia). The validation parameters for HPLC-DAD analysis are given in Table S1 (Supplementary Materials).

### 4.3. Determination of the Antioxidant Activity of Extracts

The antioxidant activity of the extracts obtained as described in Section 2.1 was evaluated based on their ability to scavenge the free radicals DPPH (2,2-diphenyl-1-picrylhydrazyl) and ABTS (2,2′-azino-bis(3-ethylbenzothiazoline-6-sulfonic acid)) and to reduce the Fe(III)–2,4,6-tripyridyl-s-triazine complex in the FRAP assay, according to [82]. Trolox solutions were used as standards in all three assays. The assay results were expressed as mg of Trolox equivalents per gram of dry weight. The absorbance of the reaction mixtures in the antioxidant activity assays was measured using a CLARIOstar microplate reader (BMG Labtech, Ortenberg, Germany).

### 4.4. Determination of the Antimicrobial Activity of Extracts

The agar-well diffusion method was used to determine antimicrobial activity [83]. Prior to the assay, 1.0 mL of each ethanol extract was dried using a vacuum concentrator. The dry extract was dissolved in 5% aqueous dimethyl sulfoxide (DMSO) to a final extract concentration of 10 mg mL^−1^. Sterile Petri dishes were placed on a horizontal surface and filled with 20 mL of 2% Hottinger agar (pH 7.2–7.4) to obtain a layer 4–5 mm thick. Using a sterile spatula, 0.1 mL of a suspension of test microorganisms (1.5 × 10^8^ CFU/mL) was evenly distributed over the agar surface, and the plates were dried. Wells 15 mm in diameter were then cut at a distance of 2.5 cm from the center of the Petri dish and positioned equidistant from one another. Test extracts (100 µL; equivalent to 1 mg of dried extract per well) were placed in the wells. The Petri dishes were incubated at 37 °C without inversion to maintain a horizontal position and ensure the formation of clear circular zones. After 24 h, the diameter of the growth inhibition zone around each well was measured. using millimeter paper, including the diameter of the well itself. An inhibition zone diameter of more than 19 mm indicated high antimicrobial activity, 15–19 mm indicated moderate activity, and less than 15 mm indicated a lack of activity. The following strains were used in the study: *Pseudomonas aeruginosa* ATCC 9027, *Staphylococcus aureus* ATCC 6538P, *Candida albicans* ATCC 10231, and *Bacillus cereus* ATCC 10702. A 5% aqueous DMSO solution was used as the negative control (100 µL per well). Fluconazole (10 µg per well) was used as the positive control in the antifungal assay, and kanamycin (10 µg per well) was used as the positive control in the antibacterial assay.

### 4.5. Determination of the Inhibitory Activity of Extracts Against α-Amylase and α-Glucosidase

The inhibitory activity assays were performed according to the protocol described in [84]. Prior to the assay, 1.0 mL of each ethanol extract was dried using a vacuum concentrator. The dry extract was dissolved in 5% aqueous DMSO to a final extract concentration of 10 mg mL^−1^.

α-Amylase (from *Aspergillus oryzae*, Sigma Aldrich, St. Louis, MO, USA) was dissolved in phosphate-buffered saline (PBS) solution (pH 6.9). The extract was added to the enzyme solution, and the mixture was incubated at 37 °C for 10 min. The final concentration of the extract in the reaction mixture was 2 mg mL^−1^. A 0.5% starch solution was then added, and the mixture was incubated at 37 °C for 5 min. Subsequently, a solution of 3,5-dinitrosalicylic acid was added, and the reaction mixture was incubated at 85 °C for 20 min. After cooling, the absorbance of the solutions was measured at 540 nm.

α-Glucosidase (from *Saccharomyces cerevisiae*, Sigma Aldrich, St. Louis, MO, USA) was dissolved in PBS buffer (pH 6.9). The extract was added to the enzyme solution, and the mixture was incubated at 37 °C for 10 min. The final concentration of the extract in the reaction mixture was 2 mg mL^−1^. Thereafter, 20 mM p-nitrophenyl-α-D-glucopyranoside (NPG, Sigma Aldrich, St. Louis, MO, USA) was added. The samples were incubated at 37 °C for 20 min. The absorbance was measured at 405 nm.

Acarbose was used as the positive control for determining the inhibitory activity of the extracts against α-amylase and α-glucosidase. The final concentration of acarbose in the reaction mixtures was 0.1 mg mL^−1^. A 5% aqueous DMSO solution was used as the control for calculating the percentage inhibition. The percentage inhibition of enzyme activity was calculated using the following formula:

Inhibition % = [(A_Control_ − A_Sample_)/A_Control_] × 100.

### 4.6. Statistical Analysis

In the course of the study, each plant species was collected and analyzed in four biological replicates corresponding to four experimental plots within the collection site. Each biochemical assay was performed in three technical replicates. Because variation among technical replicates was small, statistical analysis was performed using only biological replicates (*n* = 4). Values in the figures and tables are reported as means with their standard deviations (mean ± SD). Differences among samples were evaluated using one-way analysis of variance (ANOVA), with subsequent pairwise comparisons performed using Tukey’s test. Statistical significance was defined as *p* ≤ 0.05. Prior to ANOVA, data were tested for normality using the Shapiro–Wilk test and for homogeneity of variances. The results of these tests did not violate the evaluation by assumptions of ANOVA. Associations between phenolic compound content and antioxidant activity were examined by calculating Pearson’s correlation coefficients. Normalized means of the measured variables were used as input for cluster analysis and heat map generation, with Euclidean distance used to quantify similarity. All data processing and graphical presentation were carried out using Origin 2019b (OriginLab Corporation, Northampton, MA, USA).

## 5. Conclusions

Among the studied populations of species of the genera *Persicaria*, *Polygonum*, and *Fallopia*, extracts of *P. hydropiper*, *P. maculosa*, and *P. lapathifolia* appear to be the most promising sources of phenolic compounds, particularly ellagic acid and its derivatives, as well as compounds with antioxidant, antibacterial and α-amylase-/α-glucosidase-inhibitory activities. To characterize the phytochemical composition of the studied species more precisely, further experiments are required, involving extraction with solvents of varying polarity, fractionation of the extract, isolation of purified individual compounds, and identification based not only on UV spectra but also on mass spectrometry and nuclear magnetic resonance spectroscopy. Additionally, the potential applications of the studied species as food or medicinal raw materials are currently supported only by in vitro studies involving single-dose administration and non-mammalian enzymes. For their safer and more effective use, further studies are required, including compound-level validation, dose-dependence studies, mechanistic studies, in vivo confirmation, pharmacokinetic evaluation, and clinical investigation. The grouping of *P. hydropiper*, *P. maculosa*, and *P. lapathifolia* into a single cluster based on biochemical characteristics also supports the potential of phytochemical analysis as an auxiliary tool for refining, adjusting, and reinforcing existing systematic approaches. This study compared populations of species belonging to the genera *Persicaria*, *Polygonum*, and *Fallopia* grown under identical conditions. Broader conclusions at the level of the taxon would nonetheless require additional research covering several populations of each species and multiple growing seasons.

## Figures and Tables

**Table 1:** Quantification of individual phenolic compounds in different plant species of the genera *Persicaria*, *Polygonum,* and *Fallopia* using an HPLC-DAD system.

Compounds (Retention Time, Min)	*P. lapathifolia*	*P. hydropiper*	*P. maculosa*	*P. amphibia*	*P. minor*	*P. bistorta*	*P. aviculare*	*F. convolvulus*
Phenolic acids								
Gallic acid (4.0)	1.10 ± 0.13	1.35 ± 0.13	1.04 ± 0.09	2.42 ± 0.28	0.66 ± 0.08	0.15 ± 0.02	0.38 ± 0.03	-
3,4-Dihydroxybenzoic acid (protocatechuic acid) (6.0)	-	0.0057 ± 0.0004	0.0068 ± 0.0005	0.0657 ± 0.0050	0.0353 ± 0.0055	0.0577 ± 0.0055	0.0082 ± 0.0005	0.0330 ± 0.0050
Chlorogenic acid (7.8)	-	-	-	-	0.094 ± 0.008	-	0.160 ± 0.016	3.58 ± 0.25
Caftaric acid (9.0)	-	-	-	0.152 ± 0.013	-	-	-	1.10 ± 0.13
Caffeic acid (10.8)	-	0.082 ± 0.008	0.080 ± 0.007	-	1.67 ± 0.22	3.67 ± 0.32	0.257 ± 0.025	2.22 ± 0.24
Ferulic acid (16.8)	-	-	-	-	-	-	-	0.075 ± 0.007
Ellagic acid (19.0)	12.18 ± 1.51	11.39 ± 1.32	10.38 ± 1.19	1.23 ± 0.16	-	-	-	-
*Ellagic acid derivatives* ^1^	5.32 ± 0.59	2.66 ± 0.29	5.09 ± 0.52	1.25 ± 0.11	-	-	-	-
Flavonoids								
Catechin (10.0)	-	-	-	-	-	0.422 ± 0.040	0.485 ± 0.028	0.206 ± 0.028
Quercetin 3-β-D-galactoside (hyperoside) (19.2)	-	-	-	1.06 ± 0.13				
Quercetin 3-β-D-glucoside (isoquercitrin) (19.9)	0.748 ± 0.094	1.32 ± 0.13	0.564 ± 0.049	4.10 ± 0.38	2.89 ± 0.18	1.08 ± 0.12	0.511 ± 0.058	8.64 ± 0.89
Quercetin (36.7)	0.088 ± 0.010	0.178 ± 0.022	-	0.191 ± 0.025	0.112 ± 0.011	-	-	0.192 ± 0.023
*Quercetin derivatives* ^1^	10.30 ± 1.08	11.60 ± 1.46	9.89 ± 1.19	8.24 ± 0.88	5.10 ± 0.65	0.570 ± 0.068	6.12 ± 0.65	3.99 ± 0.40
Kaempferol 3-O-glucoside (astragalin) (25.9)	1.66 ± 0.22	1.60 ± 0.18	1.03 ± 0.10	0.238 ± 0.019	1.91 ± 0.17	0.228 ± 0.027	5.48 ± 0.61	0.333 ± 0.015
*Kaempferol derivatives* ^1^	0.309 ± 0.038	0.401 ± 0.040	0.227 ± 0.026	-	0.418 ± 0.036	1.80 ± 0.22	2.33 ± 0.26	-
Morin (29.2)	-	-	-	-	-	-	-	0.517 ± 0.028
Hesperetin (46.2)	2.09 ± 0.19	1.71 ± 0.10	1.04 ± 0.15	-	-	-	-	-
Luteolin derivatives (16.3)	-	-	-	-	0.941 ± 0.083	0.214 ± 0.020	-	0.844 ± 0.049
*Apigenin derivatives* ^1^	-	-	-	-	0.967 ± 0.079	0.580 ± 0.043	-	-
*Scutellarein derivatives* ^1^	-	-	-	-	5.06 ± 0.39	-	-	-

^1^ Compounds identified based on UV spectra and quantified using a standard with the same aglycone are indicated in italics.

**Table 2:** Antioxidant activity of extracts from plants of the genera *Persicaria*, *Polygonum,* and *Fallopia*.

Plant Species	AOA (ABTS) ^1^, mg TE g^−1^	AOA (DPPH), mg TE g^−1^	AOA (FRAP), mg TE g^−1^
*P. lapathifolia*	243.5 ± 19.2 b ^2^	159.2 ± 10.2 bc	175.8 ± 10.2 ab
*P. hydropiper*	244.7 ± 14.4 b	188.0 ± 13.9 a	125.884 ± 7.3 c
*P. maculosa*	336.9 ± 18.6 a	211.4 ± 12.6 a	190.7 ± 13.2 a
*P. amphibia*	254.2 ± 3.8 b	140.4 ± 9.3 bcd	102.1 ± 1.9 d
*P. minor*	229.2 ± 8.8 b	134.7 ± 4.3 cd	86.2 ± 5.4 de
*P. aviculare*	129.9 ± 14.5 d	121.0 ± 3.6 c	64.3 ± 3.9 e
*P. bistorta*	182.7 ± 14.4 c	128.6 ± 4.5 d	83.9 ± 5.9 de
*F. convolvulus*	261.8 ± 16.3 b	163.2 ± 2.9 b	161.3 ± 9.7 b

^1^ AOA (ABTS)—antioxidant activity determined by the ABTS (2,2′-azino-bis(3-ethylbenzothiazoline-6-sulfonic acid)) assay; AOA (DPPH)—antioxidant activity determined by the DPPH (2,2-diphenyl-1-picrylhydrazyl) assay; AOA (FRAP)—ferric reducing antioxidant power; TE—Trolox equivalents. ^2^ Different letters indicate significant differences between plant species according to Tukey’s post hoc test (*p* ≤ 0.05; *n* = 4).

**Table 3:** Antimicrobial activity of extracts from plants of the genera *Persicaria*, *Polygonum*, and *Fallopia*.

Plant Species/Controls	Inhibition Zone, mm			
	*S. aureus* (Gram-Positive)	*B. cereus* (Gram-Positive)	*P. aeruginosa* (Gram-Negative)	*C. albicans* (Fungus)
*P. lapathifolia*	21.0 ± 1.0	20.7 ± 0.6	18.7 ± 0.6	N.D. ^1^
*P. hydropiper*	23.7 ± 0.6	23.3 ± 0.6	21.3 ± 0.6	N.D.
*P. maculosa*	25.0 ± 1.0	21.0 ± 1.0	17.3 ± 0.6	N.D.
*P. amphibia*	21.0 ± 1.0	20.7 ± 0.6	18.7 ± 0.6	N.D.
*P. minor*	19.3 ± 0.6	19.7 ± 0.6	19.3 ± 0.6	N.D.
*P. aviculare*	19.0 ± 1.0	18.7 ± 0.6	17.3 ± 0.6	N.D.
*P. bistorta*	15.0 ± 0.0	17.6 ± 0.6	17.3 ± 0.6	N.D.
*F. convolvulus*	19.0 ± 0.0	18.7 ± 0.6	17.3 ± 0.6	N.D.
Kanamycin	36.3 ± 1.2	29.1 ± 0.8	34.8 ± 1.0	–
Fluconazole	–	–	–	28.0 ± 1.0
5% aqueous DMSO solution	N.D.	N.D.	N.D.	N.D.

^1^ N.D.—not detected.

## Data Availability

The original contributions presented in this study are included in the article. Further inquiries can be directed to the corresponding authors.

## Supplementary Materials

The following supporting information can be downloaded at https://www.mdpi.com/article/10.3390/plants15213333/s1, Table S1: HPLC-DAD validation parameters.
